# A Narrative Review on Toxidromes in the Psychiatric Population: Implications for Overdose Prevention

**DOI:** 10.3390/jcm14176160

**Published:** 2025-08-31

**Authors:** Sanjukta Dutta, Adela Georgiana Buciuc, Patrick Barry, Vanessa Padilla

**Affiliations:** 1 Miller School of Medicine, University of Miami, Miami, FL 33136, USA; sxd1323@miami.edu (S.D.); vpadilla@med.miami.edu (V.P.); 2 Jackson Health System, University of Miami, Miami, FL 33136, USA; patrick.barry@jhsmiami.org

**Keywords:** suicide, mental illness, psychiatry, public health, poisoning, drug overdose, anticholinergic poisoning, serotonin syndrome, neuroleptic malignant syndrome, psychotropic drugs

## Abstract

Individuals with severe mental illness face a substantially higher risk of suicide compared with the general population, with drug overdose representing one of the most common and potentially lethal methods. This narrative review explores toxidromes frequently encountered in psychiatric populations, such as opioid, anticholinergic, and serotonergic toxicity, highlighting the clinical presentation in intentional overdose. Emphasis is placed on clinical recognition, antidote-based treatment, and systems-level strategies for the prevention of lethal overdose. We conducted a comprehensive literature search of PubMed, Google Scholar, and Web of Science for English-language articles using combinations of the following keywords: mental disorders; persons with psychiatric disorders; drug overdose; poisoning; serotonin syndrome; neuroleptic malignant syndrome; anticholinergic agents/poisoning; cholinergic antagonists/poisoning; psychotropic drugs/adverse effects; substance-related disorders; drug-related side effects and adverse reactions; polypharmacy; suicide, attempted; emergency service, hospital. By embedding toxidrome awareness into routine emergency and psychiatric practice, we aim to expedite treatment and improve patient outcomes.

## 1. Introduction

Toxidromes are defined as “constellations of toxic effects comprising a set of clinical fingerprints for a group of toxic chemicals,” according to the U.S. Department of Health and Human Services [1]. They serve as clinical tools to rapidly identify toxic exposures based on characteristic symptom patterns. In both emergency medicine and psychiatric settings, early recognition of these syndromes can enable timely, potentially life-saving interventions, including the administration of antidotes and the initiation of supportive care [1,2,3,4,5].

Suicide is a major contributor to premature mortality worldwide and a leading cause of death among adolescents and young adults [6,7,8]. Methods vary by availability, with self-poisoning among the most common methods utilized globally, alongside hanging and firearms [6,7,9,10]. In high-income countries, poisoning deaths often involve prescribed and over-the-counter medicines, frequently in combination, such as antidepressants, benzodiazepines, opioids, and non-opioid analgesics; in many low- and middle-income countries, pesticide ingestion predominates [6,9,11,12]. In a national, coroner-based study, approximately one-quarter of suicides involved poisoning, and psychotropic and sedative polypharmacy was common in poisoning deaths [12]. In a review of more than 400,000 suicide attempts by drug overdose, opioids were implicated in 47.8% of fatal cases, followed by benzodiazepines and antidepressants [13].

Severe mental illness (SMI) confers a markedly elevated suicide risk and distinct method profiles. A 2024 systematic review and meta-analysis found higher odds of drug overdose among those with major depressive disorder and substantial method differences by diagnosis [14]. For example, higher rates of jumping as a method of suicide were observed in people with schizophrenia or bipolar disorder compared with people without SMI [14]. Among individuals recently discharged from psychiatric inpatient care, fatal poisonings, particularly involving psychotropic medications, accounted for more than half of unnatural deaths, and intentional self-poisoning carried a higher relative risk than violent methods [15]. The National Violent Death Reporting System data also links mental disorders and substance use disorders with an increased likelihood of poisoning as a suicide method [7].

Disproportionate toxidrome risk in psychiatric populations reflects interacting clinical and systemic drivers. These include a higher likelihood of intentional overdose during acute crises; ready access to high-risk medications and complex regimens that raise iatrogenic toxicity; impaired insight, impulsivity, and cognitive deficits that compromise safe use; and discontinuous care—especially at transitions—undermining monitoring, adherence, and rapid intervention [2,3,4,9,10,15,16].

These patterns strain emergency and inpatient services and reveal gaps in mental health care, medication management, and suicide-prevention strategies. Addressing these challenges requires targeted interventions aimed at improving access, ensuring continuity of care, and enhancing pharmacological oversight for this high-risk population.

This narrative review examines the intersection of mental illness, overdose risk, and toxidromic presentations. It summarizes the clinical features of common toxidromes encountered in medical settings, outlines diagnostic strategies and antidote-directed treatments, and highlights systems-level approaches for prevention and care coordination. By advancing point-of-care recognition and standardized response pathways in emergency and psychiatric practice, this review aims to enable timelier interventions and reduce preventable morbidity and mortality among people living with mental illness.

## 2. Materials and Methods

A literature search was conducted using PubMed, Google Scholar, and Web of Science databases. The search included English-language articles published between 2005 and May 2025 and used combinations of the following MeSH keywords: mental disorders; persons with psychiatric disorders; drug overdose; poisoning; serotonin syndrome; neuroleptic malignant syndrome; anticholinergic agents/poisoning; cholinergic antagonists/poisoning; psychotropic drugs/adverse effects; substance-related disorders; drug-related side effects and adverse reactions; polypharmacy; suicide, attempted; emergency service, hospital. A total of 259,102 records were found using this search strategy, of which approximately 36,800 records were screened, and 300 full texts were reviewed.

Inclusion criteria were studies that addressed adult patients in psychiatric or emergency settings with intentional overdose or clinically defined toxidromes. Eligible evidence included peer-reviewed original studies describing clinical features, diagnostics, management, or outcomes; systematic reviews; and authoritative clinical guidelines or toxicology references from recognized bodies, including the World Health Organization (WHO), American Heart Association (AHA), American Psychiatric Association (APA), and the U.S. Department of Health and Human Services.

Exclusion criteria were studies with pediatric-only populations; non-human or in vitro studies; non-English publications; articles lacking clinically relevant toxidrome content; and gray literature (e.g., conference abstracts, preprints, dissertations). Titles and abstracts were screened independently by two reviewers; full texts were assessed and final inclusion decisions reached by consensus, with disagreements adjudicated by a third reviewer. As the included evidence ranged from case reports to clinical guidelines, formal risk-of-bias tools were not applicable; therefore, we opted for a narrative synthesis. No preregistered protocol, quantitative synthesis, or formal risk-of-bias assessment was undertaken.

Literature was selected for inclusion based on its relevance to the following domains: the epidemiology of overdose in psychiatric populations, clinical presentations of toxidromes associated with psychotropic and illicit substances, diagnostic frameworks, therapeutic interventions, and systems-level strategies for risk mitigation. Given its non-systematic scope, we do not report PRISMA-style screening counts but instead specify search windows, databases/terms (including MeSH), inclusion/exclusion criteria, and reviewer procedures to ensure transparency. This review aims to provide a high-level synthesis to inform clinical practice and interdisciplinary collaboration.

## 3. Results

### 3.1. Epidemiology

Individuals with SMI have markedly elevated suicide risk compared with the general population [4,7,9,14,17,18,19,20]. Meta-analyses and large registries estimate pooled suicide rates around 313 per 100,000 person-years in SMI, with particularly high rates of attempted suicide in individuals with major depressive disorder and bipolar disorder [18]. Mood disorders overall confer approximately an 8- to 9-fold higher likelihood of death by suicide compared with individuals without SMI [18]. While females with SMI attempt suicide at higher rates compared with males, males experience a higher rate of death by suicide [18]. Among individuals with schizophrenia, the most severe form of psychiatric illness, suicide is the leading cause of early mortality [21]. U.S. health-system data show adjusted odds of suicide mortality of 15.0 for individuals with schizophrenia spectrum disorders and 13.2 for individuals with bipolar disorder relative to controls, with elevated risk seen across anxiety and depressive disorders as well [19]. Population studies similarly demonstrate 5- to 13-fold higher standardized mortality ratios for suicide across major psychiatric diagnoses, highest for psychotic and bipolar disorders [20]. Although firearms account for the majority of suicide deaths in the general U.S. population, poisoning is disproportionately utilized by individuals with SMI, reflecting vulnerabilities in both access and clinical context [22,23]. Moreover, the proportion of fatal outcomes from suicide attempts in this population is substantially higher than in the general population [17,24,25].

The most salient epidemiologic facts include that suicide accounts for roughly 700,000–800,000 deaths globally each year, with an estimated worldwide mortality rate of approximately 10 per 100,000 [8,9,26,27]. There exists substantial regional variation in these figures, with approximately 80% of deaths occurring in low- and middle-income countries [8]. In the United States, suicide mortality rose approximately 30–35% from 2000 to 2018 and contributed to stagnation in life expectancy; modest declines in 2019–2020 did not occur uniformly across demographic groups [26]. Suicidality extends far beyond deaths: for each suicide completed, there are approximately 20 attempts, translating to ~16 million attempts annually worldwide, and an estimated 160 million people with suicidal thoughts [8,27]. In the United States, about 10.6 million adults (4.3%) report suicidal ideation within the past year, and 1.4 million report suicide attempts in a typical year [8,27]. These data collectively underscore that suicidality is a severe, multifaceted public health crisis requiring population-level and clinical interventions.

### 3.2. Risk Factors and Clinical Relevance in Psychiatric Populations

The American Psychiatric Association (APA) emphasizes stratifying static risk factors (e.g., prior attempts, chronic severe mental illness, and medical comorbidity) and dynamic risk factors (e.g., suicidal intent/plan with accessible lethal means, intoxication, agitation, insomnia, worsening depression, treatment disengagement) when evaluating suicide risk [28].

Overdose and poisoning risk in psychiatric populations is best understood as the product of converging pathways rather than any single precipitant. Large cohort analyses demonstrate that diverse psychiatric diagnoses independently elevate poisoning risk, which further escalates with psychiatric comorbidity, indicating additive pathways of vulnerability [29,30].

Four recurrent mechanisms of vulnerability emerge: state-dependent disinhibition and impaired judgment, pharmacologic complexity, ready access to psychoactive agents, and discontinuities in care. These mechanisms interact with social and structural disadvantages to create recurrent windows of vulnerability during which high-lethality ingestions are more likely to occur and more difficult to detect early.

During acute psychiatric crises, heightened impulsivity and compromised judgment are common and strongly associated with self-injury, including overdose [21,31,32,33]. Trait impulsivity facilitates progression from suicidal ideation to attempt, particularly in adolescents and young adults [31,32,33]. Conditions such as major depressive disorder, bipolar disorder, and borderline personality disorder often involve affective lability, hopelessness, and emotional dysregulation, all of which are core risk factors for suicidality [33,34,35,36,37]. Across depressive disorders, cognitive rigidity and dichotomous thinking are consistently linked to suicidal ideation and attempts, in part due to impaired executive functions (set-shifting, cognitive flexibility) and ruminative brooding that narrow perceived options to a single “escape” and amplify hopelessness [36,37]. These thought patterns can foster the belief that suicide is the only escape from suffering. Furthermore, the presence of anhedonia and cognitive distortions can interfere with a patient’s ability to engage with protective factors like social support systems or treatment adherence. Meta-analytic and cohort findings show anhedonia predicts worse psychosocial and functional outcomes, consistent with reduced engagement with protective factors [38,39]. In borderline personality disorder, suicidal gestures may emerge in response to interpersonal conflict or perceived abandonment, often impulsive in nature, with little premeditation [40]. In psychotic disorders, persecutory delusions, auditory hallucinations, or command hallucinations may directly incite suicidal behavior, particularly when the individual lacks insight into their illness or is not receiving adequate treatment [21,41].

Psychotropic polypharmacy is frequent when treating individuals with SMI, both inpatient and outpatient, with many patients receiving multiple concurrent psychotropics alongside other medications. Large observational datasets and cohort studies show high rates of concurrent use (often ≥3 psychotropics), with antipsychotic and antidepressant combinations particularly common [42,43]. In acute psychiatric settings, a majority of patients receive multiple drugs daily, and over half receive more than one psychotropic simultaneously [42]. Community and national data similarly document substantial class and cross-class polypharmacy in severe and persistent mental illness [44,45].

Overlapping toxicodynamics are a central safety concern. This creates predictable syndromic pathways, as follows: combined opioids, benzodiazepines, gabapentinoids, and alcohol predispose to the opioid toxidrome with respiratory depression; cumulative anticholinergic load from tricyclic antidepressants, paroxetine, low-potency antipsychotics, diphenhydramine, and related agents increases the likelihood of hyperthermic anticholinergic delirium; and combining antidepressants with linezolid, tramadol, dextromethorphan, stimulants, or triptans raises the risk of serotonin syndrome. Narrow therapeutic index agents like lithium and QT-prolonging combinations add additional hazard, particularly around recent dose escalations, medication switches, or when multiple prescribers are involved without thorough reconciliation [46].

Access to means further lowers the threshold for impulsive ingestion [47]. Ready availability of prescribed and over-the-counter medications enables rapid, high-dose exposures before clinical intervention is possible [16,47].

Social and structural determinants such as loneliness, perceived burdensomeness, stigma, unemployment, housing instability, and limited access to care erode engagement and adherence, attenuating the buffering effect of outpatient care [48,49,50,51,52]. These pressures are magnified by systems-level discontinuities, such as limited access to crisis services, inconsistent medication management across settings, and especially during transition periods following ED or psychiatric discharge, when delayed follow-up and unclear clinical ownership are associated with repeated mixed ingestions and late recognition of evolving toxidromes [53,54].

Co-occurring substance use disorders are also prevalent among individuals with SMI and may contribute to both the method and lethality of suicide attempts [55]. Substances such as alcohol, opioids, synthetic cannabinoids, and stimulants not only have the ability to induce psychotic episodes but also exacerbate impulsivity [56,57,58]. These substances may be used to potentiate the effects of prescribed psychotropics, sometimes intentionally as a means of self-harm [59,60,61]. The pharmacologic complexity of these cases increases the risk of toxidrome development and complicates clinical management. Recent data from the National Center for Drug Abuse Statistics (NCDAS) highlight the severity of this intersection: 96,700 drug overdose deaths occurred in one year in the U.S. (from March 2020 to March 2021), with opioids implicated in more than 72% of these fatalities [11]. Among the most lethal substances, synthetic opioids continue to drive a sharp rise in overdose mortality, with the overall national overdose death rate at 216 per 100,000 residents [11]. These patterns highlight the critical need for integrated mental health and substance use interventions to mitigate suicide risk.

Beyond clinical and pharmacologic factors, fragmented care systems also contribute to suicide vulnerability. Individuals may face systemic barriers that increase suicide risk, such as limited access to crisis services, poor coordination between providers, medication mismanagement, and delayed interventions during critical times of vulnerability.

### 3.3. Comparison of Major Toxidromes in Psychiatry

Table 1 provides a comparative overview of common toxidromes relevant to psychiatric practice, with emphasis on pathogenesis, clinical features, and evidence-based management strategies. It is intended as a practical reference for clinicians.

1. *Anticholinergic Toxidrome*

The anticholinergic toxidrome is a well-characterized clinical syndrome resulting from inhibition of muscarinic acetylcholine receptors. It commonly arises after an overdose or from drug interactions involving medications with antimuscarinic properties, which block the ability of acetylcholine to bind to muscarinic receptors. In psychiatric practice, this toxidrome is particularly relevant due to the frequent use of tricyclic antidepressants (TCAs), antipsychotics, antihistamines, and antiparkinsonian agents, all of which can cause central and peripheral anticholinergic effects [2,3,16,62,65].

Common agents associated with this syndrome include atropine, diphenhydramine, scopolamine, hyoscyamine, amitriptyline, phenothiazines, and antiparkinsonian medications, including benztropine and trihexyphenidyl [16,62,65].

Clinical features of this syndrome occur due to widespread inhibition of parasympathetic nervous system activity. Patients may present with agitation, visual and auditory hallucinations, stereotyped picking or grabbing movements, mydriasis, tachycardia, dry mucous membranes, warm and flushed skin, urinary retention, decreased bowel sounds, and hyperthermia [3,4,62,65]. Severe toxicity may result in delirium, seizures, or coma [3,4,62,65].

The widely cited mnemonic “mad as a hatter, blind as a bat, hot as a hare, dry as a bone, red as a beet” is clinically useful in recognizing the anticholinergic toxidrome [65]. Diagnosis of anticholinergic toxicity relies on clinical recognition of its constellation of findings, supplemented by a focused medication history and exclusion of other toxidromes with overlapping features.

Management of anticholinergic toxicity is largely supportive, with agitation and seizures typically treated using benzodiazepines [2,3,4,65]. In cases of moderate to severe central toxicity manifesting as delirium, physostigmine may be considered, as it is a reversible cholinesterase inhibitor that crosses the blood–brain barrier and can overcome the effects of anticholinergics [64,65]. The use of physostigmine is controversial in cases where TCA overdose is the etiology of anticholinergic toxicity, due to the risk of precipitating asystole or seizures in patients with cardiac conduction abnormalities; therefore, a toxicologist should be consulted prior to its initiation [62,63,64].

Early identification and treatment of anticholinergic toxicity is critical. Individuals living with SMI who present with acute agitation, delirium, or autonomic instability should be evaluated for this toxidrome, particularly in the context of polypharmacy or recent medication changes.

2. *Cholinergic Toxidrome*

The cholinergic toxidrome results from overstimulation of muscarinic and nicotinic acetylcholine receptors due to inhibition of acetylcholinesterase, the enzyme responsible for breaking down acetylcholine [2,3,4,62,68,69]. This syndrome most commonly occurs following exposure to organophosphates or carbamate insecticides, but can also result from overdose of therapeutic cholinesterase inhibitors used to treat conditions such as Alzheimer’s disease or myasthenia gravis [68,84]. Although less frequently encountered in psychiatric populations, cholinergic toxicity remains a relevant consideration in cases of undifferentiated poisoning or polypharmacy.

Common offending agents include organophosphates (e.g., parathion, malathion), carbamates (e.g., carbaryl, physostigmine), and medications such as donepezil, rivastigmine, and galantamine [62,68,69]. Organophosphates phosphorylate acetylcholinesterase through an irreversible bond, thus permanently inactivating the enzyme; in contrast, carbamates form a reversible bond, which inhibits the enzyme temporarily, leading to short-lived toxicity lasting approximately 24 to 48 h [68,69].

Clinical features reflect both muscarinic and nicotinic receptor overstimulation. Muscarinic effects typically manifest as increased secretions and smooth muscle activity, such as excessive salivation, increased tear production, frequent urination, abdominal cramping, diarrhea, vomiting, pupil constriction, narrowing of the airways, and increased bronchial secretions; additional signs include bradycardia, diaphoresis, and hypotension [67,68,69]. Nicotinic effects may include fasciculations of striated muscle, cramping, weakness, and flaccid paralysis, including of the diaphragm in severe cases [67,68,69]. Both muscarinic and nicotinic receptors exist in the brain, thus contributing to combined central nervous system effects such as confusion, seizures, or coma in severe cases [62,69,85]. Most cases of death due to cholinergic toxicity are attributed to respiratory collapse or seizures [69].

The diagnosis of cholinergic toxicity is clinical and should be suspected in any patient presenting with a combination of cholinergic signs, especially when there is a history of exposure to insecticides or cholinesterase inhibitors [67,69]. Miosis, bradycardia, and bronchorrhea are particularly helpful diagnostic clues. In cases with clinical suspicion and no known exposure, a urine test can be used to detect organophosphate metabolites, and an acetylcholinesterase assay may be used for confirmatory testing [69]. In psychiatric patients, the cholinergic toxidrome may mimic other syndromes such as catatonia or serotonin toxicity, making a thorough exposure and medication history essential.

Management of cholinergic toxicity involves immediate decontamination of clothing, initiation of supportive care, and administration of antidotes [69]. Atropine, a competitive muscarinic antagonist, is the first-line treatment for cholinergic crisis, as it acts rapidly to prevent acetylcholine from binding at muscarinic receptors [67,68,69]. Although atropine has no effect on nicotinic receptors, it has shown efficacy in preventing lethal respiratory collapse [69]. Glycopyrrolate, another antimuscarinic agent, has limited central nervous system penetration and is not typically used as monotherapy but may serve as an adjunct to atropine in select cases [67,68,69,70]. Oxime therapies such as pralidoxime (2-PAM) may be used to reactivate acetylcholinesterase, even after exposure to organophosphates; these are most effective when administered early, ideally within the first few hours of exposure [62,67,69,70]. Benzodiazepines should be used for seizures or agitation, and airway support is often necessary in cases with bronchorrhea or respiratory muscle paralysis [67,69,70].

Early recognition and aggressive management are critical to improving outcomes in cholinergic toxicity [67,69]. Although rare in psychiatry, clinicians should maintain awareness of this toxidrome when evaluating patients with pinpoint pupils, respiratory distress, or excessive secretions, particularly in the context of polypharmacy or overdose involving cognitive-enhancing agents.

3. *Opioid Toxidrome*

The opioid toxidrome is a commonly encountered clinical syndrome characterized by central nervous system (CNS) depression, respiratory depression, and miosis [2,62,66,86]. It results from activation of mu-opioid receptors, which leads to suppression of brainstem respiratory centers and decreased sympathetic tone [62,66,87,88]. This toxidrome is especially relevant in psychiatric populations due to the high prevalence of co-occurring opioid use disorder in patients with SMI, as well as the increasing incidence of stimulant substances being adulterated with opioids such as fentanyl [55,89].

Agents involved with this syndrome include prescription opioids such as oxycodone, hydrocodone, hydromorphone, morphine, methadone, and buprenorphine, as well as illicit opioids including heroin and synthetic analogs such as fentanyl and carfentanil [2,62,66,89]. The latter group has contributed substantially to the rising number of fatal overdoses because of their high potency and rapid onset of respiratory suppression [88,89].

Clinical features of opioid toxicity typically include decreased level of consciousness, bradypnea or apnea, miosis (classically described as pinpoint pupils), bradycardia, hypotension, and hypothermia [2,3,62,66,86]. In severe cases, non-cardiogenic pulmonary edema and respiratory failure may occur [88,90]. Although miosis is a characteristic finding, it may be absent in polysubstance overdoses or in patients with hypoxia due to advanced toxicity. Hyporeflexia with flaccid muscle tone may also be observed [62].

Diagnosis of opioid toxicity is clinical and should be considered in any patient presenting with altered mental status and hypoventilation, particularly when there is a known or suspected history of opioid use [62,66]. Although not universally present, the triad of CNS depression, respiratory depression, and miosis is suggestive [2,62,66]. Confirmatory testing, such as a urine drug screen, may support the diagnosis but should not delay treatment.

Management begins with airway stabilization and ventilatory support [66]. The opioid antagonist naloxone is the treatment of choice and can be administered intranasally, intramuscularly, or intravenously depending on the clinical situation [66]. Naloxone reverses opioid-induced respiratory and CNS depression by competitively binding to mu receptors [66,88,91]. Naloxone should be titrated to restore adequate respiratory effort rather than full arousal, as rapid reversal may precipitate acute withdrawal, agitation, or cardiovascular instability [91]. In cases involving long-acting opioids or potent synthetic analogs, repeated administrations or continuous infusions of naloxone may be required due to its short half-life [92].

In psychiatric settings, opioid toxicity may present either as an isolated overdose or as part of a polysubstance ingestion. Prompt recognition of this toxidrome is critical, especially in patients with a history of substance use, mood disorders, or prior suicide attempts.

4. *Sedative-Hypnotic Toxidrome*

The sedative-hypnotic toxidrome is characterized by generalized central nervous system (CNS) depression resulting from the enhancement of gamma-aminobutyric acid (GABA) activity at GABA-A receptors [71,72]. This toxidrome is most commonly associated with benzodiazepines, barbiturates, ethanol, and non-benzodiazepine hypnotics like Z-drugs [2,62,71,72]. In psychiatric populations, sedative-hypnotic agents are frequently prescribed for anxiety, insomnia, or agitation, and are also commonly co-ingested in intentional overdoses [62,71,72].

Common agents include benzodiazepines (e.g., diazepam, lorazepam), barbiturates (e.g., phenobarbital), ethanol, meprobamate, and the so-called “Z-drugs” such as zolpidem and zaleplon. These substances act via similar mechanisms, facilitating chloride influx through GABA-A receptors and leading to neuronal hyperpolarization and CNS depression [72].

The sedative-hypnotic toxidrome is characterized by CNS depression with normal or decreased vital signs. Core findings include somnolence to coma, slurred speech, ataxia/incoordination, unsteady gait, nystagmus, impaired attention and memory (often anterograde amnesia), and respiratory depression with loss of airway reflexes in severe cases [71,72,93,94]. Pupils are typically normal or slightly constricted; skin is usually normal; bowel sounds are normal or decreased [72,93]. Respiratory depression is less common with isolated benzodiazepine overdose but may be pronounced in co-ingestion with other CNS depressants such as opioids or alcohol [71,72]. Complications include trauma from falls and motor vehicle crashes, hypoventilation, hypoxemia/hypercarbia, and coma; lethality rises with co-ingestants (opioids, ethanol) [71,93].

Diagnosis is clinical and should be considered in any patient with unexplained CNS depression, especially in the setting of a known psychiatric history or access to sedative medications. While serum drug levels can aid confirmation and diagnosis, treatment should not be delayed for laboratory results.

Management of sedative-hypnotic toxidrome is primarily supportive. The American Heart Association (AHA) emphasizes airway compromise and hypoxemia as mechanisms of death in benzodiazepine overdose and recommends standard airway and ventilatory support as first-line management [5]. Flumazenil may reverse sedation, but carries seizure and dysrhythmia risk in tolerant patients or mixed overdoses [5,73,95]. It should be avoided in patients with suspected co-ingestion of tricyclic antidepressants or in chronic benzodiazepine users due to the risk of precipitating seizures or acute withdrawal [73,95]. Dosing should be cautiously titrated with readiness to treat seizures. The AHA advises that in undifferentiated coma or mixed overdoses, the potential for flumazenil to precipitate refractory withdrawal, seizures (including in patients with epilepsy), and dysrhythmias outweighs its benefit; harms are amplified with co-ingested pro-convulsants (notably cyclic antidepressants) and hypoxia, and reversal may be incomplete in mixed sedative exposures [5]. The AHA emphasizes use only in low-risk scenarios (iatrogenic oversedation, pediatric exploratory ingestion) after excluding benzodiazepine tolerance and dangerous co-ingestants [5].

Patients presenting with sedative-hypnotic toxicity often have overlapping psychiatric or substance use disorders. A high index of suspicion and careful attention to medication history are essential for early identification and appropriate management.

5. *Sympathomimetic Toxidrome*

The sympathomimetic toxidrome results from excessive stimulation of adrenergic receptors by endogenous or exogenous catecholamines [2,3,4,62,74]. This syndrome is commonly associated with the use or overdose of central nervous system stimulants, such as amphetamines, cocaine, and synthetic cathinones [74]. In psychiatric populations, this toxidrome is particularly relevant due to the prevalence of stimulant misuse, comorbid substance use disorders, and access to prescribed psychostimulants for conditions such as attention-deficit/hyperactivity disorder (ADHD) [74,96].

The most common agents include amphetamines, methamphetamine, cocaine, methylphenidate, synthetic cathinones (“bath salts”), and sympathomimetic decongestants such as pseudoephedrine, phenylephrine, and ephedrine [74,75]. These agents exert their effects by increasing synaptic concentrations of norepinephrine, dopamine, and serotonin through enhanced release, reuptake inhibition, or both.

Clinical features are marked by autonomic hyperactivity and CNS excitation. Typical signs include hypertension, tachycardia, hyperthermia, agitation, paranoia, hallucinations, mydriasis, tremor, diaphoresis, and gastrointestinal distress such as nausea, vomiting, or abdominal pain [2,74].

The cardiovascular complications associated with sympathomimetic drugs are diverse and range from hemodynamic perturbations to life-threatening events [97]. The American Heart Association (AHA) emphasizes that acute sympathomimetic toxicity can cause tachycardia, hypertension, hyperthermia-related metabolic derangements, and can precipitate malignant arrhythmias and cardiac arrest [5]. Vasospasm-mediated ischemia can lead to myocardial infarction even with angiographically normal coronaries, and stress (takotsubo) cardiomyopathy has been reported [5]. The AHA also notes dose-related blood pressure and heart rate elevations with therapeutic and over-the-counter sympathomimetics (e.g., pseudoephedrine, ephedrine), which may be clinically consequential in comorbid patients [98].

Chronic or high-dose exposure to amphetamines/methamphetamines and related stimulants has been associated with dilated cardiomyopathy, heart failure, myocardial infarction, aortic dissection, pulmonary edema, arrhythmias, and sudden death [5,74]. Importantly, methamphetamine-associated cardiomyopathy may be at least partially reversible with cessation and guideline-directed therapy, per the AHA Scientific Statement on specific dilated cardiomyopathies [99].

Catecholamine excess induces coronary vasospasm, supply–demand mismatch, mitochondrial dysfunction, oxidative stress, calcium overload, and myocyte death, underpinning ischemia, cardiomyopathy, and arrhythmogenesis [100]. AHA statements on drug-induced arrhythmias also implicate stimulants (amphetamine/methamphetamine/MDMA) in atrial and ventricular arrhythmias via catecholamine excess [5]. ADHD stimulants produce small mean increases in heart rate and blood pressure; rare reports include arrhythmia, nonischemic and takotsubo cardiomyopathy, and sudden death, though large-scale causal risk remains uncertain [97,101].

Diagnosis is clinical and based on the characteristic constellation of signs and symptoms. The presence of severe agitation, dilated pupils, tachycardia, and hyperthermia should prompt consideration of this toxidrome. Unlike anticholinergic toxicity, patients with sympathomimetic toxicity typically present with diaphoresis rather than dry skin.

Management of sympathomimetic toxicity is centered on supportive care and sedation. Benzodiazepines are the first-line treatment to reduce agitation, lower sympathetic outflow, and prevent seizures [74]. For patients with severe hypertension or tachyarrhythmias, alpha2-adrenergic agonists (such as dexmedetomidine or clonidine) or direct vasodilators (such as nitroprusside) may be required [5,74]. Beta-blockers should be avoided as monotherapy, particularly nonselective agents, due to the risk of unopposed alpha-adrenergic stimulation and worsening vasoconstriction [5]. In cases of hyperthermia, active cooling measures should be initiated promptly.

Sympathomimetic toxicity frequently occurs in the context of polysubstance use, often complicating the clinical picture [102]. In psychiatric patients, prompt recognition and management are essential to prevent cardiovascular, neurologic, and metabolic complications.

6. *Neuroleptic Malignant Syndrome*

Neuroleptic malignant syndrome (NMS) is a severe, life-threatening toxicity resulting from dopamine receptor antagonism in the basal ganglia, hypothalamus, and brainstem, most commonly associated with overdose or adverse effects from typical and atypical antipsychotics [2,3,4,62,76,77]. Psychiatric patients are particularly vulnerable to NMS due to the widespread use of antipsychotic medications for conditions such as schizophrenia, bipolar disorder, and acute agitation, as well as for off-label indications including mood disorders and behavioral disturbances [76,77].

Common causative agents include first-generation (typical) antipsychotics such as haloperidol, fluphenazine, and chlorpromazine; second-generation (atypical) agents such as risperidone and olanzapine; and phenothiazine derivatives used as antiemetics (e.g., prochlorperazine, promethazine) [76,77]. These agents exert antagonistic effects at dopamine D2 receptors in the central nervous system, leading to characteristic movement-related and autonomic side effects, commonly referred to as extrapyramidal symptoms (EPS), or in severe cases, life-threatening NMS. It has been proposed that preexisting central dopaminergic hypoactivity, coupled with changes to the dopamine system from repeated stress exposure, is the primary mechanism of NMS [76].

The most common clinical features of EPS include acute dystonia, trismus (jaw clenching), oculogyric crisis (involuntary eye movements), akathisia (inner restlessness), parkinsonism, bradykinesia, ataxia, and tremor. In some cases, hypotension may occur due to alpha-adrenergic blockade [76,77]. While EPS may be uncomfortable for patients, they are not typically life-threatening [76,77]. In contrast, NMS is a life-threatening response to dopamine antagonism and is distinguished by lead-pipe rigidity, altered mental status, autonomic dysregulation (including labile blood pressure and hyperthermia), and elevated creatine kinase [76,77]. These effects may be misinterpreted as worsening psychiatric symptoms, which can delay appropriate intervention [16,76,77]. NMS is typically associated with high-potency dopamine antagonists, psychomotor agitation, rapid dose titration, and can also occur after abrupt withdrawal of dopaminergic medications [76].

Diagnosis of NMS is clinical and based on the presence of extrapyramidal signs and autonomic instability in the context of recent or ongoing antipsychotic exposure. Differentiation from other toxidromes, such as serotonin syndrome, is essential and can be guided by neuromuscular findings [78]. Clonus and hyperreflexia, for example, are more characteristic of serotonergic toxicity, whereas rigidity and bradyreflexia suggest dopaminergic blockade [78].

Management of mild to moderate EPS includes discontinuation of the offending agent and administration of anticholinergic medications such as benztropine or diphenhydramine [76,77,78]. In more severe cases, particularly those resembling anticholinergic toxicity, physostigmine may be used cautiously under toxicology consultation [76,77,78]. In the setting of NMS, dopamine agonists such as bromocriptine or amantadine are first-line treatments, along with dantrolene for muscle rigidity and hyperthermia [76,77,78]. Supportive care, including hydration, electrolyte correction, and intensive monitoring, is critical for recovery [16,62,76,77,78].

Recognizing NMS is essential for the timely reversal of symptoms and prevention of life-threatening complications. Psychiatric populations are especially at risk of this toxidrome due to chronic antipsychotic use, polypharmacy, and delays in diagnosis when symptoms mimic psychiatric deterioration [76,77].

7. *Serotonergic Toxidrome (Serotonin Toxicity)*

Serotonin syndrome is a potentially life-threatening toxidrome caused by excessive serotonergic activity in the central and peripheral nervous systems. It is most often associated with the use or combination of two or more serotonergic agents, including antidepressants, opioids, and other medications with serotonergic properties [2,3,4,16,62,79,81,82]. Psychiatric patients are particularly at risk due to polypharmacy, treatment-resistant mood disorders, and the use of multiple serotonergic agents across therapeutic classes [81,82].

Common agents implicated in serotonin syndrome include selective serotonin reuptake inhibitors (SSRIs), serotonin–norepinephrine reuptake inhibitors (SNRIs), monoamine oxidase inhibitors (MAOIs), tricyclic antidepressants (TCAs), and atypical antidepressants such as trazodone and mirtazapine [79,80,81,82]. Additional contributors include opioid analgesics like meperidine, tramadol, and fentanyl; serotonergic anxiolytics such as buspirone; over-the-counter agents like dextromethorphan; and some synthetic cannabinoids and herbal products [79,80,81,82].

Clinical features of serotonin syndrome typically develop rapidly after a change in dose or the addition of a new serotonergic agent [82]. Symptoms span three domains: mental status changes (agitation, confusion), autonomic instability (hyperthermia, hypertension, tachycardia, diaphoresis), and neuromuscular abnormalities (tremor, hyperreflexia, inducible or spontaneous clonus, ocular clonus, and myoclonus) [81,82]. Hyperreflexia and clonus are key distinguishing features from NMS and anticholinergic toxicity [78]. Gastrointestinal symptoms such as diarrhea may also be present [80,81,82].

Diagnosis of serotonin syndrome is clinical and often guided by the Hunter Serotonin Toxicity Criteria, which require the presence of a serotonergic agent and specific neuromuscular findings such as inducible or spontaneous clonus in combination with other core features of serotonin toxicity [81,103]. As there are no biomarkers for serotonin syndrome, laboratory testing is nonspecific for diagnosis and is primarily used to assess complications such as rhabdomyolysis, acidosis, or renal injury [81].

Management of mild to moderate serotonin syndrome involves immediate discontinuation of all serotonergic medications and supportive care [79,80,81,82]. Benzodiazepines are first-line agents for sedation, agitation, and seizure prevention [79,80,81,82]. For moderate to severe cases, cyproheptadine, a serotonin 2A antagonist, may be administered orally or via a nasogastric tube [81,82,104]. Alpha2-adrenergic agonists such as dexmedetomidine or clonidine may be used as adjunctive therapies in intensive care settings to manage autonomic symptoms when benzodiazepines alone are insufficient [80,83]. Hyperthermia in serotonin syndrome is believed to occur due to overstimulation of postsynaptic serotonin receptors, resulting in increased central catecholamine release and sympathetic nervous system activation [105,106,107]. As this is a centrally driven thermogenesis, antipyretics such as acetaminophen and ibuprofen are largely ineffective for treating hyperthermia caused by serotonin syndrome [79,80,81,82]. In cases of significant hyperthermia, aggressive external cooling and intensive care monitoring are essential [81,82].

Due to overlapping features with other syndromes, such as NMS, and pre-existing psychiatric symptoms, serotonin syndrome may be underrecognized. Given the widespread use of serotonergic medications, clinicians should maintain a high index of suspicion when encountering patients with agitation, hyperreflexia, or autonomic instability.

### 3.4. Clinical Approach to Overdoses

**Figure 1:**
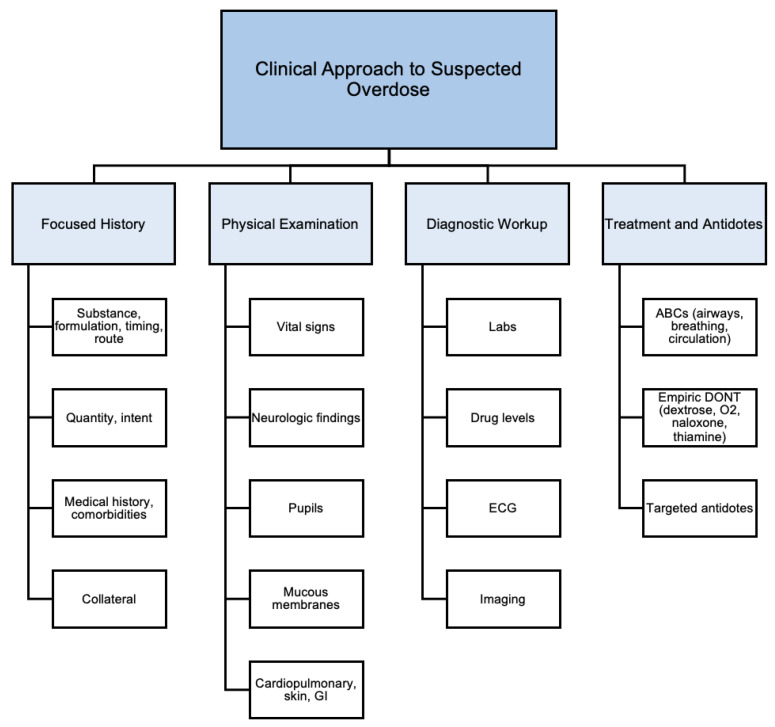
Clinical approach to suspected overdose.

A systematic and efficient approach is essential when evaluating a patient with suspected overdose [108]. Psychiatric populations often present with diagnostic uncertainty, mixed ingestions, or nonspecific symptoms, thus underscoring the need for rapid identification of toxidromes and prompt initiation of supportive care [108]. A comprehensive clinical assessment typically spans four domains: focused history, physical examination, diagnostic workup, and treatment [2,3,62,108,109]. Please see Figure 1 below for a framework outlining the clinical assessment of overdose.

1. *Focused History*

A thorough history is crucial when evaluating potential ingestion and should focus on the “what,” “when,” and “how much” of any substances involved. Key elements include identifying the specific agents and formulations—whether prescription, over-the-counter, or illicit—as well as the time and route of ingestion. It is important to determine the quantity of substance ingested, along with the individual’s intent, such as whether the event was a suicide attempt, recreational use, or therapeutic error. A comprehensive assessment should also consider any pre-existing medical conditions, particularly hepatic, renal, or cardiac disease, which may influence metabolism and toxicity. Additionally, evaluating access to medications within the home or care facility can provide critical context for risk assessment.

Collateral information from family, emergency medical services, medication lists, or pill bottles can be invaluable in clarifying the clinical context of an overdose, particularly when patients have impaired insight, delirium, psychosis, or other conditions that limit the reliability of their history [108]. Accordingly, history-gathering should be triangulated from collateral sources available at the point of care. High-yield elements include accounts from emergency personnel or bystanders to establish the time of last known well; pill counts and medication bottles retrieved from the scene; blister packs and pharmacy labels indicating dosing instructions and fill dates; and pharmacy or prescription drug monitoring program data to confirm recent dispenses and identify multiple prescribers. Family members, roommates, or caregivers can provide details about the patient’s baseline mental status, recent stressors, and access to over-the-counter products. Documenting the reliability of each source and explicitly linking collateral data to the exposure timeline both improve diagnostic accuracy and reduce anchoring on a primary psychiatric explanation. Clinicians should also obtain and verify documentation of prior suicide attempts and/or overdose history, as recurrent suicide attempts are associated with a substantially higher risk of severe or fatal overdose [34].

2. *Physical Examination*

The physical examination is a critical bedside tool that helps guide the differential diagnosis and often points toward a specific toxidrome. Particular attention should be given to vital signs, as abnormalities such as hyperthermia, bradycardia, or hypotension can offer important diagnostic clues [108]. Neurologic findings, including level of consciousness, presence or absence of seizures, agitation, rigidity, clonus, and hyperreflexia, can help distinguish between various toxic syndromes; the Glasgow Coma Scale (GCS) can be used to identify patients in need of immediate intubation or resuscitation [108]. Pupillary size and reactivity, as well as the moisture of mucous membranes, provide further insight into autonomic involvement, which may be helpful in differentiating opioid toxicity from the sympathomimetic toxidrome [108]. Nystagmus may be present in patients with a variety of toxic ingestions, including phenytoin, phencyclidine, carbamazepine, lithium, ethanol, barbiturates, and sedative-hypnotics [108]. Skin findings such as diaphoresis, dryness, flushing, or cyanosis can be telling features of specific poisonings. Additionally, gastrointestinal signs like altered bowel sounds or urinary retention may be present, and the cardiopulmonary exam may reveal tachyarrhythmias, wheezing, or rales suggestive of pulmonary edema, all of which contribute valuable information toward narrowing the diagnosis.

The ability to recognize consistent patterns, such as the combination of miosis, bradypnea, and CNS depression in opioid toxicity or hyperthermia with clonus and agitation in serotonin syndrome, facilitates early presumptive diagnosis and intervention [108].

3. *Diagnostic Workup*

Laboratory and imaging studies play a key role in confirming suspected toxic overdoses, identifying complications, and monitoring the patient’s response to treatment. A standard initial workup should include measurement of electrolytes, renal function, and liver enzymes, along with serum glucose to detect hypoglycemia or hyperglycemia [108]. An arterial or venous blood gas is essential to assess acid-base status, while creatine kinase should be checked to screen for rhabdomyolysis. Additional important labs include a coagulation panel and lactate, which can indicate systemic toxicity or poor perfusion. Acetaminophen and salicylate levels should be obtained in all cases of suspected overdose—even if not initially suspected—due to their potential for severe morbidity. In women of childbearing age, a urine pregnancy test is necessary; overdoses in pregnant patients often occur for suicidal or abortifacient reasons, especially in psychiatric patients or in settings where intimate partner violence is suspected [108,110]. Urine drug screens and urinalysis, though limited in scope, may aid in the identification of commonly abused substances and provide additional diagnostic clues [108]. An electrocardiogram (ECG) should also be performed to assess cardiac effects such as QT prolongation, QRS widening, or bradyarrhythmias, which can guide both diagnosis and management. In cases involving altered mental status, seizures, or head trauma, neuroimaging such as a noncontrast CT scan of the head may be warranted [108]. A chest radiograph can be obtained to evaluate for aspiration pneumonitis or pulmonary edema.

4. *Treatment and Antidote Administration*

The foundation of overdose management is supportive care, including airway stabilization, oxygenation, intravenous fluids, and correction of electrolyte or acid-base disturbances [2,108]. Close cardiac and respiratory monitoring is essential in all moderate to severe intoxications.

In patients presenting with undifferentiated altered mental status, the empiric administration of low-risk, high-benefit interventions is recommended while the underlying cause is being determined. These interventions include dextrose to address potential hypoglycemia, oxygen to correct hypoxia or respiratory depression, naloxone if opioid overdose is suspected, and thiamine, particularly in individuals who are malnourished or have a history of alcohol use [2,3,62,108].

Once a specific toxidrome is identified, targeted antidotes should be promptly administered. Naloxone remains the antidote of choice for opioid toxicity, while flumazenil may be considered for benzodiazepine overdose only in carefully selected patients due to the risk of seizures. Physostigmine can be used in cases of severe anticholinergic toxicity, and cyproheptadine is indicated for serotonin syndrome. In cholinergic poisoning, such as from organophosphates, atropine and pralidoxime are the mainstays of treatment. For neuroleptic malignant syndrome, dantrolene and bromocriptine may be used to counteract the hyperthermia and dopamine blockade.

Activated charcoal may be considered within one hour of ingestion in alert patients with a protected airway to reduce drug absorption. However, it has not been shown to significantly improve patient outcomes [108,111]. Whole bowel irrigation with polyethylene glycol is indicated in patients who have ingested particular substances, such as lithium, drug-filled packets, heavy metals, or sustained-release products [108]. Lipid emulsion therapy and hemodialysis are reserved for selected poisonings with lipid-soluble or dialyzable agents [3,62,108].

Timely recognition of toxidromes, coupled with supportive and antidotal therapy, significantly improves outcomes in overdose management [108]. Multidisciplinary collaboration with toxicology, psychiatry, and pharmacy is strongly recommended in complex or unclear cases.

### 3.5. Distinguishing Toxidromes from Psychiatric Syndromes

Differentiating substance-induced alterations in mental status from primary psychiatric conditions depends on acuity, autonomic physiology, and neuromuscular findings, which primary psychiatric disorders rarely reproduce in a consistent cluster. Toxidromes typically emerge within minutes to hours after a plausible exposure, whereas exacerbations of mood or psychotic illness progress over days to weeks and are not tightly linked to a single dose.

Autonomic features often provide the strongest basis for distinction. Patterns such as hyperthermia; marked tachycardia or hypertension; diaphoresis versus pronounced mucosal dryness; altered bowel motility; urinary retention; and characteristic pupillary changes (miosis in opioid toxicity, mydriasis in anticholinergic or sympathomimetic states) each increase the likelihood of a toxicologic process. Neuromuscular findings further refine the differential. Generalized or ocular clonus with diffuse hyperreflexia supports serotonin toxicity. “Lead-pipe” rigidity with bradykinesia and elevated creatine kinase suggests neuroleptic malignant syndrome. Agitated delirium with hot, dry skin, mydriasis, urinary retention, and diminished bowel sounds is characteristic of anticholinergic poisoning. Hypoventilation with pinpoint pupils strongly indicates opioid toxicity.

Since phenomenology can overlap, such as when anticholinergic delirium mimics acute psychosis or manic agitation resembles sympathomimetic toxicity, clinicians should maintain a low threshold for bedside testing. High-yield evaluations include point-of-care glucose, core temperature, venous blood gas, and capnography when hypoventilation is suspected, as well as early electrocardiography (QRS widening in tricyclic antidepressant toxicity and marked QTc prolongation with certain antipsychotics or methadone). Therapeutic probes can both clarify diagnosis and accelerate treatment. Examples include naloxone for suspected opioid toxicity, carefully monitored physostigmine for anticholinergic delirium in appropriate settings, and a lorazepam challenge when malignant catatonia is considered. Routine broad urine toxicology panels are at best supportive and should not delay empiric, syndrome-directed care when a toxidrome is suspected.

### 3.6. Overdose Prevention and Longitudinal Systems-Level Interventions

The long-term management of toxidromes in psychiatric populations must extend beyond acute stabilization to encompass longitudinal strategies that reduce the likelihood of recurrent overdose, minimize medication-related harm, and promote sustained recovery. While acute care focuses on toxidrome recognition and reversal, lasting risk reduction requires addressing the underlying behavioral, pharmacologic, and systemic contributors to toxic exposures. Psychiatric patients often face complex treatment regimens, fragmented access to care, and limited support during transitions between inpatient and outpatient settings, all of which heighten their vulnerability to toxic overdose [15,19]. A systems-based approach is essential to mitigate these risks, and we propose a four-pronged approach for providers that integrates the following: safer prescribing practices, robust follow-up protocols, interdisciplinary collaboration, and targeted public health interventions. When implemented collectively, these strategies can reduce the incidence of medication toxicity and overdose while improving long-term outcomes for individuals living with SMI.

1. *Restricting Access to High-Risk Medications*

Means restriction has emerged as a cornerstone of suicide prevention, aiming to reduce the availability of substances and tools commonly used in suicide attempts. A growing body of evidence supports the effectiveness of this approach in reducing suicide risk across diverse settings and populations [112,113,114,115,116,117,118]. A recent systematic review of suicide prevention strategies found that when patients are faced with a lack of access to their preferred method of suicide (e.g., firearms, chemicals), most do not turn to alternative methods, making restriction a valuable strategy capable of motivating behavioral change [117].

Risk mitigation strategies can be implemented by reconciling prescriptions appropriately, limiting dispensed quantities of high-risk agents, using blister or unit-dose packaging, employing secure storage, and arranging disposal of unused medications [66,112,113,114,115,116,117,118]. Medication reconciliations should extend beyond simple list consolidation to include interaction checks, assessment of cumulative anticholinergic and serotonergic burden, and consolidation of prescribing and pharmacy oversight where feasible [112,113]. Psychotropic agents such as tricyclic antidepressants, monoamine oxidase inhibitors, and long-acting opioids carry significant risks when misused. Therefore, clinicians should consider safer alternatives when possible, especially for patients with a history of suicide attempts or substance use disorders [117]. In high-risk cases, supervised administration or long-acting injectable formulations may further reduce the likelihood of overdose [119]. Structured suicide safety assessments should always be paired with medication-specific means of safety.

2. *Enhanced Patient Education and Discharge Planning*

Patients treated for overdose and patients with SMI are at increased risk of recurrent admissions and carry a significantly increased rate of mortality, particularly within the first days and weeks after discharge [120]. Therefore, timely outpatient follow-up, education, and communication between inpatient and community providers are essential to ensure safe care transitions [117]. Scheduling psychiatric or primary care appointments within 72 h (goal ≤ 7 days) is shown to reduce recurrent presentations [116]. Structured safety planning through the use of digital tools such as telepsychiatry visits, automated reminders, and virtual check-ins may improve post-discharge adherence [117,121]. These practices are consistent with the World Health Organization’s LIVE LIFE strategy, which emphasizes early identification, continuous monitoring, and follow-up of individuals affected by suicidal behavior [6].

When opioids are present in the regimen or involved in the event, naloxone should be co-prescribed, and patients, along with their household members, should be educated on overdose recognition, prevention, and naloxone use [113,114]. Alcohol and stimulant use disorders should be addressed with counseling and facilitated referrals, with pharmacotherapy arranged when appropriate [115]. Standardizing these measures, alongside compact toxidrome-guided recognition and antidote pathways, connects point-of-care diagnosis to prevention and can accelerate treatment.

3. *Pharmacist Involvement and Collaborative Care Models*

Pharmacists are critical to the safe prescribing and monitoring of psychotropic medications, particularly in patients with complex regimens. Integrating pharmacists into multidisciplinary psychiatric care teams has been shown to improve medication adherence, enhance patient education, and support safe prescribing practices [122,123,124]. Collaborative care models that include psychiatry, primary care, behavioral health, and pharmacy are especially effective for patients with co-occurring mental and physical illnesses, improving both safety and clinical outcomes [118,125,126]. Despite the increased upfront costs associated with establishing collaborative care models, there is growing evidence that they lead to long-term health care savings and higher patient satisfaction, with the added benefit of adapting to the unique mental health concerns of specific populations, such as students, geriatric patients, women, and individuals with substance use disorders [125,126].

4. *Public Health and Policy Interventions*

Public health interventions can play a central role in reducing the incidence of overdose and suicide by improving awareness, reducing stigma, and encouraging recognition of risk within communities. The World Health Organization identifies effective measures such as community-based naloxone distribution, medication take-back programs, and safe storage campaigns to limit access to lethal means [6]. These large-scale measures can inform state- and community-level policies that directly influence patient outcomes. Public education campaigns, training for healthcare providers, and responsible media reporting on suicide can reduce stigma and enhance early intervention [127,128]. Online suicide education programs can further enhance suicide literacy among participants without ready access to care, reduce self-stigma, and promote self-efficacy to seek support in psychologically difficult situations [128]. Increasing the availability of accessible online public health resources may broaden reach and improve engagement in at-risk populations. In parallel, real-time public surveillance systems that track trends in poisoning and self-harm are critical for guiding resource allocation and informing the development of responsive, data-driven interventions [129,130].

## 4. Limitations

This selective narrative review has several limitations. We did not preregister a protocol, conduct a formal risk-of-bias assessment, or perform a meta-analysis, as substantial heterogeneity in study designs, exposure definitions, and outcomes precluded meaningful quantitative synthesis. Our search was limited to English-language, peer-reviewed sources and excluded gray literature, introducing potential publication and language bias. Because part of the search relied on Google Scholar, which yields non-replicable result sets, we report approximate screening counts rather than definitive study totals.

## 5. Conclusions

Toxidromes represent a clinically essential yet often underutilized framework for identifying and managing toxicologic emergencies in psychiatric populations. Individuals with SMI are at elevated risk for overdose due to a convergence of factors, such as widespread access to high-risk medications, co-occurring substance use disorders, dynamic factors, and fragmented systems of care. As this narrative review has demonstrated, these vulnerabilities are compounded by the diagnostic complexity of mixed ingestions and the overlapping presentations of psychiatric syndromes and toxidromes.

Accurate and timely recognition of toxidromes requires both structured clinical assessment and familiarity with characteristic symptom patterns. This narrative review provides a practical, point-of-care framework for overdose management in psychiatric settings. We connect psychiatric risk pathways to the toxicologic syndromes clinicians most often face and translate them into actions that fit emergency department and consultation-liaison psychiatry workflows. Diagnostic red flags include abrupt onset after a plausible exposure and autonomic instability out of proportion to primary psychiatric illness, with signature findings that accelerate decision-making. Opioid toxicity presents with miosis and hypoventilation; anticholinergic toxicity with hot, dry, agitated delirium, mydriasis, urinary retention, and decreased bowel sounds; and serotonin toxicity with spontaneous or inducible clonus, hyperreflexia, and hyperthermia. An early electrocardiogram helps detect tricyclic-related QRS widening.

Prevention and systems strategies should be equally actionable. These include limiting the quantity of medications dispensed, ensuring secure storage, and facilitating proper disposal of unused medications. At every transition of care, rigorous medication reconciliation should be performed, including checks with the Prescription Drug Monitoring Program (PDMP) and screening for potential drug interactions. When opioids are prescribed, naloxone should be co-prescribed and accompanied by patient education. Antidotes for specific toxidromes (e.g., naloxone, carefully selected physostigmine, or cyproheptadine) should be rapidly administered when indicated, alongside appropriate supportive care. Treatment for substance use disorders should be initiated or bridged from the emergency department when appropriate. Rapid follow-up after discharge should be arranged, ideally within 72 h, with a goal of no more than 7 days post-discharge, and warm handoffs should be used to ensure continuity of care. Prescribing and pharmacy oversight should be consolidated, with pharmacists integrated into psychiatric care teams whenever possible. Embedding these steps can shorten the time to antidote-directed treatment and reduce preventable morbidity and mortality among people living with SMI. On a broader scale, public health interventions and policy reforms informed by surveillance data and grounded in harm reduction principles are essential. Bridging the gap between psychiatry and toxicology offers the opportunity to reduce preventable morbidity and mortality, improve medication safety, and foster a model of care that is more resilient, compassionate, recovery-oriented, evidence-based, person-centered, and responsive to the needs of individuals with mental illness.

## Figures and Tables

**Table 1:** Overview of common toxidromes in psychiatry.

Toxidrome	Pathogenesis	Common Agents	Clinical Features	Diagnosis	Management
**Anticholinergic**	Muscarinic acetylcholine receptor blockade causes central and peripheral Ach inhibition	Atropine, diphenhydramine, hyoscyamine, TCAs, phenothiazines, benztropine, trihexyphenidyl, scopolamine	**Dry skin**, mydriasis, urinary retention, ileus, delirium, **hyperthermia**, **tachycardia**, flushed skin, and hallucinations. Severe: **delirium**, **seizures**, coma	Clinical: dry skin, mydriasis, **altered mental status**; **absent diaphoresis** (differentiates from sympathomimetic)	Supportive care, benzodiazepines; **physostigmine** in severe cases [2,3,62,63,64,65]
**Opioid**	Mu-opioid receptor agonism	Heroin, morphine, oxycodone, fentanyl, carfentanil, methadone	**Miosis**, **respiratory depression**, bradycardia, hypotension, coma	Clinical: **classic triad**—miosis, respiratory depression, loss of consciousness	**Naloxone**, airway support [2,3,62,66]
**Cholinergic**	Excess acetylcholine due to acetylcholinesterase inhibition	Organophosphates, carbamates, nerve agents, physostigmine	**D**iarrhea, **U**rination, **M**iosis, **B**radycardia, **B**ronchorrhea, **E**mesis, **L**acrimation, **S**alivation, **S**weating	Clinical: muscarinic + nicotinic symptoms, bradycardia, wheezing, secretions	**Atropine**, pralidoxime, benzodiazepines [2,3,62,67,68,69,70]
**Sedative-Hypnotic**	Potentiation of GABA-A receptor activity	Benzodiazepines, barbiturates, zolpidem, ethanol	CNS depression, **slurred speech, ataxia**, hypotension, respiratory depression	Clinical: history + **CNS depression** without other findings (e.g., **no miosis or clonus**)	Supportive care; **flumazenil** is rarely used due to risk of seizures or arrhythmia [2,3,62,71,72,73]
**Sympathomimetic**	Excessive stimulation of adrenergic receptors via increased catecholamine release or reuptake inhibition	Amphetamines, methamphetamine, cocaine, methylphenidate, synthetic cathinones, pseudoephedrine, phenylephrine, ephedrine	**Hypertension**, **tachycardia**, **hyperthermia**, agitation, paranoia, hallucinations, mydriasis, **tremor**, diaphoresis, **seizures**, rhabdomyolysis	Clinical: agitation, mydriasis, hyperthermia, **+diaphoresis** (distinguishes from anticholinergic)	Supportive care, benzodiazepines, cooling; avoid beta-blockers alone! [2,3,5,62,74,75]
**Neuroleptic**	Dopamine D2 receptor blockade, primarily in the basal ganglia and hypothalamus	Haloperidol, fluphenazine, risperidone, olanzapine, prochlorperazine, promethazine	Mild (EPS): dystonia, tremor, bradykinesia, akathisia; Severe (NMS): **hyperthermia**, **lead-pipe rigidity**, altered mental status, **autonomic instability**	Clinical: rigidity, altered mental status, **fever**, increased creatine kinase	Stop the offending agent! EPS: benztropine or diphenhydramine. NMS: **bromocriptine**, **dantrolene**, ICU support [2,3,62,76,77,78]
**Serotonergic**	Excess serotonergic activity, particularly at 5-HT2A receptors	SSRIs, SNRIs, MAOIs, TCAs, trazodone, mirtazapine, tramadol, fentanyl, dextromethorphan, buspirone	Agitation, **clonus, hyperreflexia**, mydriasis, hyperthermia, diarrhea, tremor, altered mental status	**Hunter Criteria**: clonus + serotonergic agent; hyperreflexia and clonus are key signs	Stop all serotonergic drugs; **benzodiazepines**, cyproheptadine, cooling; ICU monitoring if severe [2,3,62,79,80,81,82,83]
